# Elucidation of a masked repeating structure of the O-specific polysaccharide of the halotolerant soil bacteria *Azospirillum halopraeferens* Au4

**DOI:** 10.3762/bjoc.12.62

**Published:** 2016-04-04

**Authors:** Elena N Sigida, Yuliya P Fedonenko, Alexander S Shashkov, Nikolay P Arbatsky, Evelina L Zdorovenko, Svetlana A Konnova, Vladimir V Ignatov, Yuriy A Knirel

**Affiliations:** 1 Institute of Biochemistry and Physiology of Plants and Microorganisms, Russian Academy of Sciences, Prospekt Entuziastov 13, Saratov 410049, Russia; 2 N. D. Zelinsky Institute of Organic Chemistry, Russian Academy of Sciences, Leninsky Prospekt 47, Moscow 119991, Russia; 3 Chernyshevsky Saratov State University, Ulitsa Astrakhanskaya 83, Saratov 410012, Russia

**Keywords:** *Azospirillum halopraeferens*, bacterial polysaccharide structure, lipopolysaccharide, O-specific polysaccharide, Smith degradation, triflic acid solvolysis

## Abstract

An O-specific polysaccharide was obtained by mild acid hydrolysis of the lipopolysaccharide isolated by the phenol–water extraction from the halotolerant soil bacteria *Azospirillum halopraeferens* type strain Au4. The polysaccharide was studied by sugar and methylation analyses, selective cleavages by Smith degradation and solvolysis with trifluoroacetic acid, one- and two-dimensional ^1^H and ^13^C NMR spectroscopy. The following masked repeating structure of the O-specific polysaccharide was established: →3)-α-L-Rha*p2Me*-(1→3)-[β-D-*Glcp*-(1→4)]-α-D-Fuc*p*-(1→2)-β-D-Xyl*p*-(1→, where non-stoichiometric substituents, an O-methyl group (~45%) and a side-chain glucose residue (~65%), are shown in italics.

## Introduction

Rhizobacteria of the genus *Azospirillum* are isolated from a wide variety of environments. Their ubiquitous distribution in nature is evidently due to the extraordinary plasticity of their genomes and the ability to form beneficial associations with plants owing to plant-growth promoting activities [1]. The plant–microbe symbiosis increases the tolerance of both partners to various environmental factors, among which soil salinity is one of the most stressful. As halotolerant plant-growth-promoting rhizobacteria are environmentally friendly and energy efficient, they attract attention as promising biotechnological agents for combating crop salinity stress [2]. These microorganisms mitigate deleterious salt stress effects by producing osmoprotectants and enzymes and inducing plant systemic resistance. Additionally, they enhance plant growth by the synthesis of phytohormones and vitamins, fixation of atmospheric nitrogen, and solubilization of soil phosphates [2–4].

Strains of the species *Azospirillum halopraeferens* are isolated from the rhizoplane of kallar grass (*Leptochloa fusca* L. Kunth), which is widely distributed in tropical and subtropical regions [5] and was successfully introduced as a pioneer plant in salt-contaminated infertile areas [6]. Being halotolerant, *A. halopraeferens* stimulates the growth of halophyte forage and oilseed crops in seawater irrigated agriculture [7]. The successful use of *Azospirillum* inoculants requires understanding the mechanisms regulating their interactions with plants at a molecular level.

A lipopolysaccharide (LPS) having an O-specific polysaccharide chain (OPS) called O antigen and capsular polysaccharide (K antigen) of *Azospirillum*, which is an extracellular form of LPS [8–9], are important for the interaction between bacteria and host plants. The cell-surface polysaccharides of *Azospirillum* are involved in overcoming of unfavorable conditions, including survival of bacteria under salinity [10–13]. Preliminary chemical data on the LPS of *A. halopraeferens* type strain Au4, including fatty acid and monosaccharide composition, have been reported [14]. As biological functions of the LPS are expected to depend on their structures, this study aimed at elucidation of the OPS structure of *A. halopraeferens* Au4.

## Results and Discussion

In our previous studies [14], a high-molecular mass OPS was obtained by degradation of the LPS of *A. halopraeferens* Au4 under mild acidic conditions in order to cleave the acid-labile linkage between the carbohydrate and glycolipid parts [15] followed by fractionation of the released water-soluble carbohydrate portion by Sephadex G-50 Superfine size-exclusion chromatography. It was demonstrated that the OPS contained 2-*O*-methyl-6-deoxyhexose, L-rhamnose (Rha), D-fucose (Fuc), D-xylose (Xyl), and D-glucose (Glc) in the ratios ~1:1.9:2.8:2.2:2.1 (detector response data). In the present work, 2-*O*-methyl-6-deoxyhexose was identified as L-Rha2Me by GLC, GLC–MS and NMR (see below).

Substitution patterns of the monosaccharides in the OPS were determined using alkylation analysis. Earlier, 2,3,4,6-tetra-*O*-methylglucose, 2,4-di-*O*-methylrhamnose, 2,4-di-*O*-methylfucose, 2-*O*-methylfucose, and 3,4-di-*O*-methylxylose were identified by GLC–MS of partially methylated alditol acetates derived after methylation of the OPS with MeI followed by hydrolysis and acetylation [14]. Therefore, the OPS contains 3-substituted Rha, 3-substituted Fuc, 3,4-disubstituted Fuc, 2-substituted Xyl, and terminal Glc. The OPS is branched with Glc in the side chain and Fuc at the branching point. In the present work, using EtI in place of MeI both 2,4-di-*O*-ethylrhamnose and 4-*O*-ethyl-2-*O*-methylrhamnose were identified. Hence, the 3-substituted Rha residue is partially 2-O-methylated in the OPS, a finding consistent with identification of Rha2Me in sugar analysis.

The ^1^H and ^13^C NMR spectra of the OPS (Supporting Information File 1) showed signals of different intensities and thus indicating a structural irregularity. Further studies revealed that its reason is a non-stoichiometric side-chain glucosylation and methylation of the main polysaccharide chain but at this stage, a straightforward structure elucidation of the OPS by NMR spectroscopy [16] was complicated.

In order to obtain oligosaccharide fragments of the OPS a Smith degradation was used, which included periodate oxidation of the vicinal hydroxy groups in the monosaccharides, mild acidic hydrolysis at the linkages of the destroyed sugar residues, and borohydride reduction of aldehyde groups before and after hydrolysis [17]. Based on the methylation analysis data (see above), elimination of the side-chain Glc and cleavage of the linkage of the destroyed 2-substituted Xyl were expected.

**Scheme 1:**
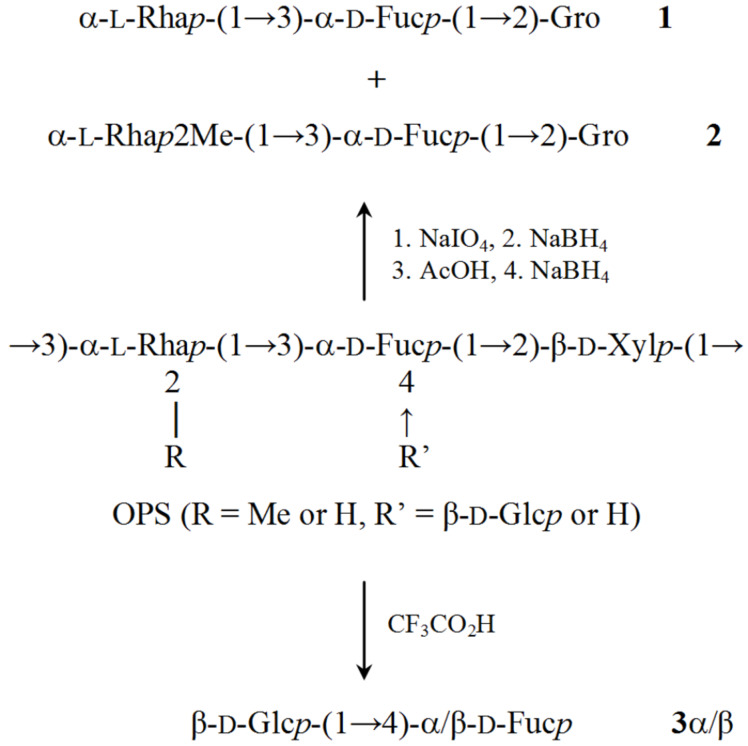
Structures of the OPS from *A. halopraeferens* Au4 and oligosaccharides obtained from the OPS by Smith degradation (**1** and **2**) and solvolysis (**3**). Gro indicates glycerol.

Fractionation of the Smith degradation products by TSK HW-40 (S) gel-permeation chromatography resulted in a mixture of the expected oligosaccharides **1** and **2** and higher molecular mass compounds. The ^1^H and ^13^C NMR (Supporting Information File 1) spectra of **1** and **2** were assigned using two-dimensional ^1^H–^1^H COSY, TOCSY, and ^1^H–^13^C HSQC experiments (Table 1). Tracing connectivities in the COSY and TOCSY spectra combined with ^3^*J*_H,H_ coupling constant data for sugar ring protons [18] revealed spin systems for glycerol (Gro) derived from the 2-substituted Xyl and *manno* (Rha)- and *galacto* (Fuc)-configurated monosaccharides (Scheme 1). The spectra of compound **2** also showed signals for a methyl group (δ_H_ 3.47–3.48, δ_C_ 59.9). The values of ^13^C NMR chemical shifts of the C-5 signals of Rha and Rha2Me at δ 70.2–70.4 and Fuc at δ 68.1 were close to published data of the corresponding α-anomers (δ 69.4 and 73.6 for α- and β-Rha, δ 67.5 and 71.9 for α- and β-Fuc, respectively [19]); therefore, these monosaccharides were α-linked.

**Table 1:** ^1^H and ^13^C NMR chemical shifts of the oligosaccharides derived from the OPS from *A. halopraeferens* Au4 by Smith degradation (**1** and **2**) and solvolysis with CF_3_CO_2_H (**3**).^a^ Gro indicates glycerol.

Residue	δ [ppm]						

	C-1	C-2	C-3	C-4	C-5	C-6	OMe
	*1-H (1a,1b)*	*2-H*	*3-H (3a,3b)*	*4-H*	*5-H*	*6-H (6a,6b)*	

Oligosaccharide **1**:							

α-L-Rha*p*- (1→	103.7	71.5	71.4	73.4	70.2	17.8	
	*5.02*	*4.08*	*3.87*	*3.48*	*3.81*	*1.29*	
→3)-α-D-Fuc*p*-(1→	99.4	68.7	78.9	73.0	68.1	16.4	
	*5.10*	*3.92*	*4.00*	*3.89*	*4.26*	*1.22*	
→2)-Gro	61.7	80.1^b^	62.4				
	*3.69, 3.80*	*3.79*	*3.69, 3.79*				

Oligosaccharide **2**:							

α-L-Rha*p*2Me-(1→	100.2	81.4	71.1	73.6	70.4	17.8	59.9
	*5.17*	*3.72*	*3.90*	*3.40*	*3.85*	*1.29*	*3.47*
→3)-α-D-Fuc*p*-(1→	99.4	68.8	79.0	73.0	68.1	16.4	
	*5.10*	*3.98*	*4.00*	*3.89*	*4.26*	*1.22*	
→2)-Gro	61.7	80.0^b^	62.4				
	*3.69, 3.80*	*3.79*	*3.69, 3.79*				

Disaccharide **3α**:							

β-D-Glc*p*-(1→	105.2	75.3	77.2	70.8	77.3	61.9	
	*4.78*	*3.47*	*3.62*	*3.51*	*3.53*	*3.84, 4.01*	
→4)-α-D-Fuc*p*	93.5	69.9	71.2	83.0	67.2	17.2	
	*5.32*	*3.95*	*4.04*	*4.17*	*4.35*	*1.36*	

Disaccharide **3β**:							

β-D-Glc*p*-(1→	105.1	75.3	77.2	70.8	77.3	61.9	
	*4.79*	*3.47*	*3.62*	*3.51*	*3.53*	*3.84, 4.01*	
→4)-β-D-Fuc*p*	97.5	73.4	74.8	82.1	71.6	17.2	
	*4.68*	*3.62*	*3.83*	*4.11*	*3.94*	*1.40*	

^a1^H NMR chemical shifts are given in italics. ^b^Assignment could be interchanged.

The ^13^C NMR signals for C-3 of Fuc in **1** and **2** shifted significantly downfield to δ 78.9–79.0 as compared with its positions in the non-substituted α-Fuc at δ 70.6 [19]. The ^13^C NMR chemical shifts for C-2–C-6 of Rha in **1** were close to those of the non-substituted monosaccharide [19], whereas in **2**, the signal for C-2 of Rha was observed in a low field at δ 81.4 evidently due to 2-*O*-methylation. Hence, Rha and Rha2Me occupied the non-reducing end in compounds **1** and **2**, respectively (Scheme 1).

The isolated higher-molecular mass compounds were separated by reverse-phase HPLC and demonstrated by ^1^H and ^13^C NMR spectroscopy (data not shown) to be dimeric by-products resulted from incomplete cleavage between the destroyed 2-substituted Xyl and the neighbouring non-methylated Rha.

As Glc was destroyed and eliminated by Smith degradation, the information about the configuration of its linkage and the site of its attachment was lost. In other to obtain a Glc-containing oligosaccharide, selective solvolysis with CF_3_CO_2_H was employed. Recently, this method has been successfully used for the structure elucidation of the O-specific polysaccharides of *Escherichia coli* (e.g. [20]). The reagent was found to cleave selectively the glycosidic linkage of 6-deoxyhexoses (Rha, Fuc), whereas the linkage of hexoses (Glc, Man, Gal) were unaffected. Solvolysis of the OPS with CF_3_CO_2_H cleaved all glycosidic linkages (Rha, Fuc, Xyl) but the Glc linkage. As a result, disaccharide **3** with Fuc at the reducing end was isolated, its structure (shown in Scheme 1) was established as described above (for assigned ^1^H and ^13^C NMR chemical shifts of **3α** and **3β** see Table 1 and Supporting Information File 1). Particularly, the β configuration of Glc followed from a relatively low-field positions of the C-5 signals at δ 77.3 in the ^13^C NMR spectrum of the disaccharide **3** as compared with the published data δ 72.3 and 76.8 for α- and β-Glc, respectively [19]. The 4-substitution of Fuc was inferred from a significantly downfield shift to δ 82.1–83.0 of the C-4 signal as compared with its positions in the non-substituted α-Fuc at δ 72.9 [19].

Combining structural data of the oligosaccharides **1–3** enabled suggestion of the general OPS structure (Scheme 1). This structure was confirmed by NMR spectroscopy, including assignment of the ^1^H and ^13^C NMR spectra (Table 2) as described above for compounds **1** and **2** and sequence analysis by two-dimensional ^1^H–^1^H ROESY and ^1^H–^13^C HMBC experiments.

**Table 2:** ^1^H and ^13^C NMR chemical shifts of the OPS from *A. halopraeferens* Au4.^a^

Residue	δ [ppm]						

	C-1 *1-H*	C-2 *2-H*	C-3 *3-H*	C-4 *4-H*	C-5 *5-H* *(5a,5b)*	C-6 *6-H* *(6a,6b)*	OMe

O-Methylated glucosylated unit:							

→3)-α-L-Rha*p*2Me-(1→	99.6	80.5	80.9	72.4	70.3	18.0	59.3
	*5.19*	*3.89*	*3.91*	*3.54*	*3.76*	*1.29*	*3.41*
→3,4)-α-D-Fuc*p*-(1→	99.5	69.6	77.3	78.5	68.3	16.8	
	*5.41*	*4.14*	*4.07*	*4.18*	*4.46*	*1.21*	
→2)-β-D-Xyl*p*-(1→	106.0	79.1	75.7	70.4	66.1		
	*4.75*	*3.44*	*3.53*	*3.67*	*3.31, 3.94*		
β-D-Glc*p*-(1→	103.5	74.7	77.3	70.9	77.3	62.0	
	*4.77*	*3.35*	*3.47*	*3.41*	*3.38*	*3.71, 3.89*	

Non-methylated glucosylated unit:							

→3)-α-L-Rha*p*-(1→	102.6	71.2	81.0	72.4	70.3	18.0	
	*5.07*	*4.22*	*3.87*	*3.64*	*3.76*	*1.29*	
→3,4)-α-D-Fuc*p*-(1→	99.5	69.6	77.3	78.5	68.3	16.8	
	*5.41*	*4.14*	*4.07*	*4.18*	*4.46*	*1.21*	
→2)-β-D-Xyl*p*-(1→	106.1	79.1	75.7	70.4	66.3		
	*4.79*	*3.44*	*3.54*	*3.66*	*3.36, 3.98*		
β-D-Glc*p*-(1→	103.5	74.7	77.3	70.9	77.3	62.0	
	*4.77*	*3.35*	*3.47*	*3.41*	*3.38*	*3.71, 3.89*	

O-Methylated non-glucosylated unit:							

→3)-α-L-Rha*p*2Me-(1→	100.0	80.5	80.9	72.3	70.2	17.9	59.4
	*5.15*	*3.89*	*3.91*	*3.54*	*3.85*	*1.27*	*3.42*
→3)-α-D-Fuc*p*-(1→	99.3	68.3	79.4	73.0	68.0	16.3	
	*5.43*	*3.94*	*3.95*	*3.88*	*4.39*	*1.19*	
→2)-β-D-Xyl*p*-(1→	106.0	79.1	75.7	70.4	66.1		
	*4.75*	*3.44*	*3.53*	*3.31*	*3.32, 3.94*		

Non-methylated non-glucosylated unit:							

→3)-α-L-Rha*p*-(1→	103.3	71.2	81.0	72.4	70.3	18.0	
	*5.00*	*4.22*	*3.87*	*3.64*	*3.76*	*1.29*	
→3)-α-D-Fuc*p*-(1→	99.3	68.3	78.9	73.0	68.0	16.3	
	*5.43*	*3.94*	*3.94*	*3.88*	*4.39*	*1.19*	
→2)-β-D-Xyl*p*-(1→	106.1	79.1	75.7	70.4	66.3		
	*4.79*	*3.44*	*3.54*	*3.66*	*3.36, 3.98*		

^a1^H NMR chemical shifts are given in italics.

The significant shifts of the Xyl C-2, Fuc C-3 and C-4, Rha and Rha2Me C-3 signals to a lower field of δ 77.3–81.0, as compared with their positions in the respective unsubstituted monosaccharides [19], indicated the modes of sugar glycosylation in the OPS. The C-2–C-6 chemical shifts of Glc were characteristic of an unsubstituted residue in the β-anomeric form [19] and thus confirmed that β-Glc occupied the terminal position in the side chain. The ROESY spectrum (Supporting Information File 1) showed the following cross-peaks between the anomeric protons and the protons at the linkage carbon atoms: Rha2Me 1-H/Fuc 3-H, Rha 1-H/Fuc 3-H, Fuc 1-H/Xyl 2-H, Xyl 1-H/Rha 3-H, Xyl 1-H/Rha2Me 3-H, and Glc 1-H/Fuc 4-H. The sequence of the sugar residues thus defined was confirmed by the HMBC spectrum (Supporting Information File 1), which displayed correlations between the anomeric protons and transglycosidic carbons: Fuc 1-H/Xyl C-2, Xyl 1-H/Rha C-3, Xyl 1-H/Rha2Me C-3, Glc 1-H/Fuc C-4, Rha2Me 1-H/Fuc C-3, Rha 1-H/Fuc C-3.

The relative integral intensities of the 1-H signals of the Rha and Rha2Me residues and 5-H signals of the 3-substituted and 3,4-disubstituted Fuc in the ^1^H NMR spectrum of the OPS indicated that the degree of O-methylation was ca. 45% and the degree of side-chain glucosylation was ca. 65%.

## Conclusion

Based on the chemical and NMR spectroscopic data, it was concluded that the OPS of *A. halopraeferens* Au4 has a masked repeating structure and consists of four types of oligosaccharide units, which differ in the presence or absence of the side-chain Glc and O-methyl group. In order to solve the intricate structure selective cleavages of the OPS by the well-known Smith degradation [17] and solvolysis with a recently introduced reagent, CF_3_CO_2_H, [20] were performed. The two cleavages afforded complementary oligosaccharides, which identification shed light on the nature of the OPS irregularity and, combined with chemical analysis and NMR spectroscopic analysis data, enabled the structure elucidation of the OPS.

Chemical modifications of the OPS, such as O-methylation or O-acetylation, often non-stoichiometric, are not uncommon for Gram-negative bacteria. They occur independently of the polymerization mechanism [21–22] and often are associated with temperate bacteriophages that bear the corresponding transferases. Such alterations in certain O-antigen structures of emerging human pathogens *Salmonella*, *Escherichia* and *Shigella* [23–25] increase the antigenic diversity and evidently are helpful for evasion from recognition by immune system cells [26]. The bacterium *Shigella flexnery* is a vivid illustration of how adding of α-D-Glc, OAc and PEtN groups at various positions of the polysaccharide backbone diversifies enormously their O-unit structures [25]. In addition, side-chain glucosylation of the OPS of these bacteria contributes to acid resistance [27] and is crucial for their permeation into the host cell [28]. Plant-associated bacteria are no exception, and the presence of non-stoichiometric substituents in the OPS has been reported for phytopathogens *Pseudomonas syringae* [29] and *Xanthomonas campestris* [30] as well as for beneficial rhizobacteria, including free-living *Azospirillum* spp. [31–35] and root-nodulating *Rhizobium* spp. [24]. Moreover, the degree of acetylation and/or methylation of the rhizobial cell surface glycans depends on the growth phase [36] and cultivation conditions (*in planta* or *ex planta*) [26]. These modifications may promote attachment of the bacteria to the roots by an increase of the hydrophobicity of their cell surface. In turn, the increase in cell hydrophobicity, leading to the formation of cell aggregates or to their attachment to soil particles, was established for several microorganisms under salt stress, including azospirilla [12], and is regarded to be a mechanism that allows bacteria to survive in hostile environment.

## Experimental

### General procedures

GLC of the alditol acetates [37] and 2-octyl glycosides [38] and GLC–MS of the partially alkylated alditol acetates [39] were performed using an Agilent 7820A GC system and an Agilent MSD 5975C instrument equipped with an HP-5ms column, respectively. GLC parameters were set and chemical modifications of the OPS into the corresponding derivatives were performed as described [35]. NMR spectra were obtained using an Avance II 600 MHz instrument (Bruker, Germany) at 30 °C in 99.95 % D_2_O using sodium 3-trimethylsilylpropanoate-2,2,3,3-*d*_4_ (δ_H_ 0.0, δ_C_ −1.6) as internal standard for calibration. Two-dimensional NMR experiments were performed using standard Bruker software. Spin lock time in TOCSY experiments and mixing time in ROESY experiments were set to 60 and 200 ms, respectively. The HMBC spectrum was recorded with a 60-ms delay for evolution of long-range couplings. Samples were prepared and other NMR parameters were set essentially as described [40].

**Bacterial growth, isolation of LPS and OPS**. *A. halopraeferens* strain Au4 (IBPPM 221) [5] was obtained from the microbial culture collection of the Institute of Biochemistry and Physiology of Plants and Microorganisms, Russian Academy of Sciences (IBPPM RAS, Saratov) and was cultivated under aerobic conditions at 41 °C in a liquid malate medium [8] supplemented with 0.09 M NaCl. In an analogous manner as described before [14,33] the cells were washed from the capsule and dried, LPS was extracted from the biomass (10 g) by the Westphal procedure [41] in a yield 7.4%, and OPS was isolated and purified by gel filtration in a yield 39% of the LPS mass.

**Selective cleavages.** Periodate oxidation of an OPS sample (50 mg) was performed as described [35]. The oxidized polysaccharide was hydrolysed with 2% AcOH at 100 °C for 2 h, reduced with NaBH_4_, desalted with an Amberlit IR-120 (H^+^-form) resin and fractionated by exclusion chromatography on TSK HW-40 (S) in 1% AcOH to yield a mixture of oligosaccharide (OS) **1** and OS **2** (7 mg) and a higher molecular mass material. The latter was fractionated by HPLC on a reverse-phase Zorbax C18 column (25 × 1 cm) in water (1 mL min^−1^) monitored with a differential refractometer (Waters, USA).

An OPS sample (25 mg) was treated with anhydrous CF_3_CO_2_H (0.5 mL) at 45 °C for 3 h. After evaporating the acid, the products were dissolved in H_2_O and fractionated by exclusion chromatography on TSK HW-40 (S) in 1% AcOH to yield an OS **3** (3 mg).

## Supporting Information

File 1 ^1^H and ^13^C NMR spectroscopy data of the O-specific polysaccharide and of the oligosaccharides **1**, **2** and **3**.
