# Supplementary material for: Elucidation of a masked repeating structure of the O-specific polysaccharide of the halotolerant soil bacteria Azospirillum halopraeferens Au4
Source: Beilstein J Org Chem. 2016 Apr 4;12:636–42. doi: 10.3762/bjoc.12.62 (PMC4902059; doi:10.3762/bjoc.12.62)
Supplement: File 1 — 1H and 13C NMR spectroscopy data of the O-specific polysaccharide and of the oligosaccharides 1, 2 and 3. [file Beilstein_J_Org_Chem-12-636-s001.pdf]

Supporting Information

for

Elucidation of a masked repeating structure of the O-specific polysaccharide of the halotolerant soil bacteria *Azospirillum halopraeferens* Au4

Elena N. Sigida[*,§,1], Yuliya P. Fedonenko[1], Alexander S. Shashkov[2], Nikolay P. Arbatsky[2], Evelina L. Zdorovenko[2], Svetlana A. Konnova[1,3], Vladimir V. Ignatov[1] and Yuriy A. Knirel[2]

Address: [1]Institute of Biochemistry and Physiology of Plants and Microorganisms, Russian Academy of Sciences, Prospekt Entuziastov 13, Saratov 410049, Russia, [2]N. D. Zelinsky Institute of Organic Chemistry, Russian Academy of Sciences, Leninsky Prospekt 47, Moscow 119991, Russia and [3]Chernyshevsky Saratov State University, Ulitsa Astrakhanskaya 83, Saratov 410012, Russia

Email*:* Elena N. Sigida - si_elena@mail.ru

*Corresponding author.

[§]Tel.: +7 8452 970444; Fax: +7 8452 970383.

^{1}H and ^{13}C NMR spectroscopy data of the O-specific polysaccharide and of the oligosaccharides 1, 2 and 3.

Content

NMR spectroscopy data of the OPS from *A. halopraeferens* Au4 and of the oligosaccharides obtained after selective cleavages of the OPS.

Scheme S1: Structures of the repeating units of the OPSs from *A. halopraeferens* Au4..…………..…..s2

Figure S1: ^{1}H NMR spectrum of the OPS……………………………………………………….s3

Figure S2: ^{13}C NMR spectrum of the OPS.……………………………………………...…….s4

Figure S3: Fragment of the ^{1}H, ^{13}C HMBC spectrum of the OPS………………………………..s5

Figure S4: Fragment of the ^{1}H, ^{1}H ROESY spectrum of the OPS………………………………..s6

Figure S5: ^{13}C NMR spectrum of the mixture of the oligosaccharides **1** and **2**…………………………s7

Figure S6: ^{1}H NMR spectrum of the mixture of the oligosaccharides **1** and **2**…………………………..s8

Figure S7: Fragments of the ^{1}H, ^{13}C HSQC spectrum of the oligosaccharide **3**………………………...s9

```
        (R)                  (F)                 (X)
→3)-α-L-Rhap-(1→3)-α-D-Fucp-(1→2)-β-D-Xylp-(1→            1
                             4
                             ↑
                             1
                          β-D-Glcp
                            (G)

      (R2Me)                 (F)                 (X')
→3)-α-L-Rhap2Me-(1→3)-α-D-Fucp-(1→2)-β-D-Xylp-(1→         2
                                4
                                ↑
                                1
                             β-D-Glcp
                               (G)

        (R')                (F')                (X)
→3)-α-L-Rhap-(1→3)-α-D-Fucp-(1→2)-β-D-Xylp-(1→            3

      (R'2Me)                (F')                (X')
→3)-α-L-Rhap2Me-(1→3)-α-D-Fucp-(1→2)-β-D-Xylp-(1→         4
```

Scheme S1: Structures of the repeating units of the OPSs from *A. halopraeferens* Au4.

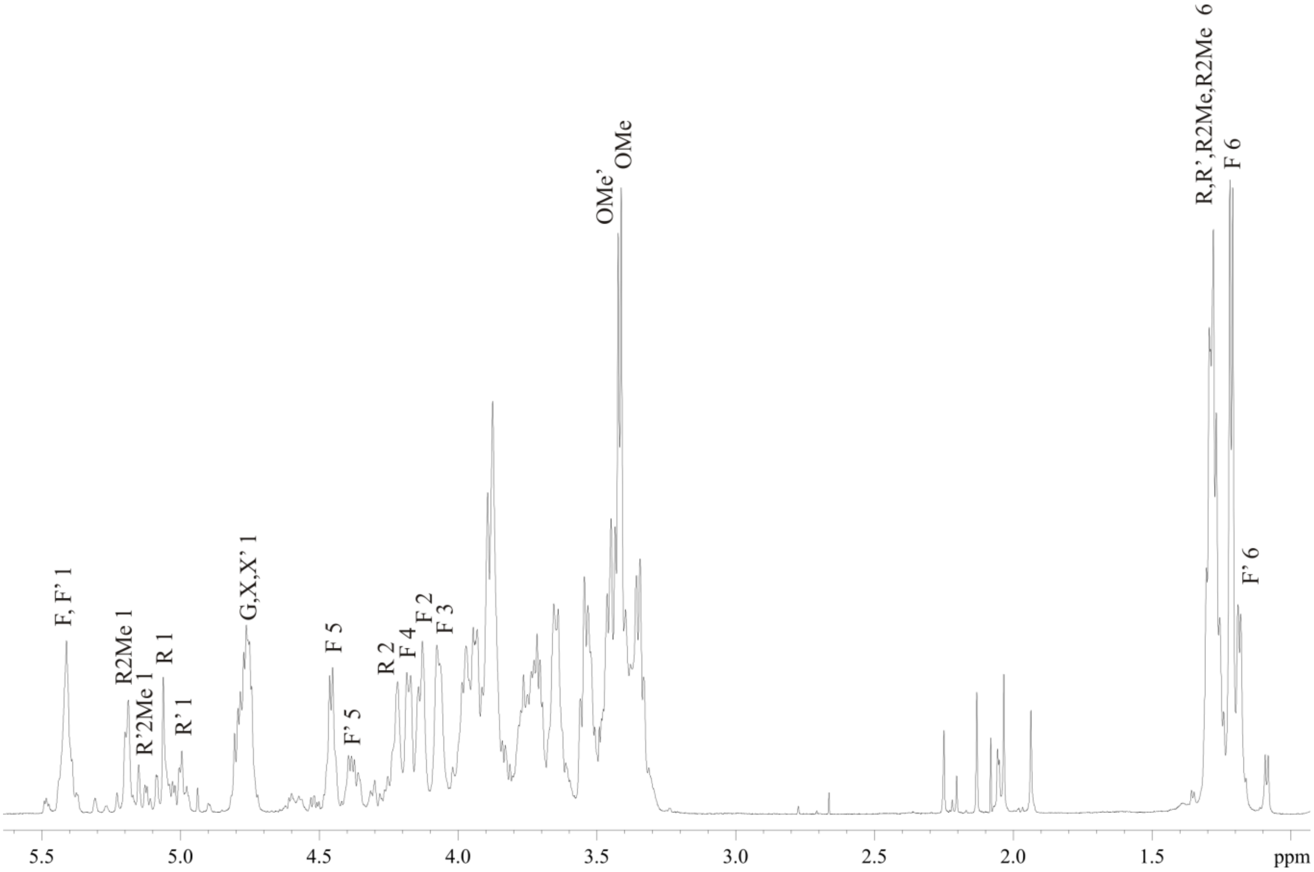

Figure S1: ^{1}H NMR spectrum of the OPS from *A. halopraeferens* Au4. Arabic numerals refer to protons in sugar residues as denoted in Scheme S1.

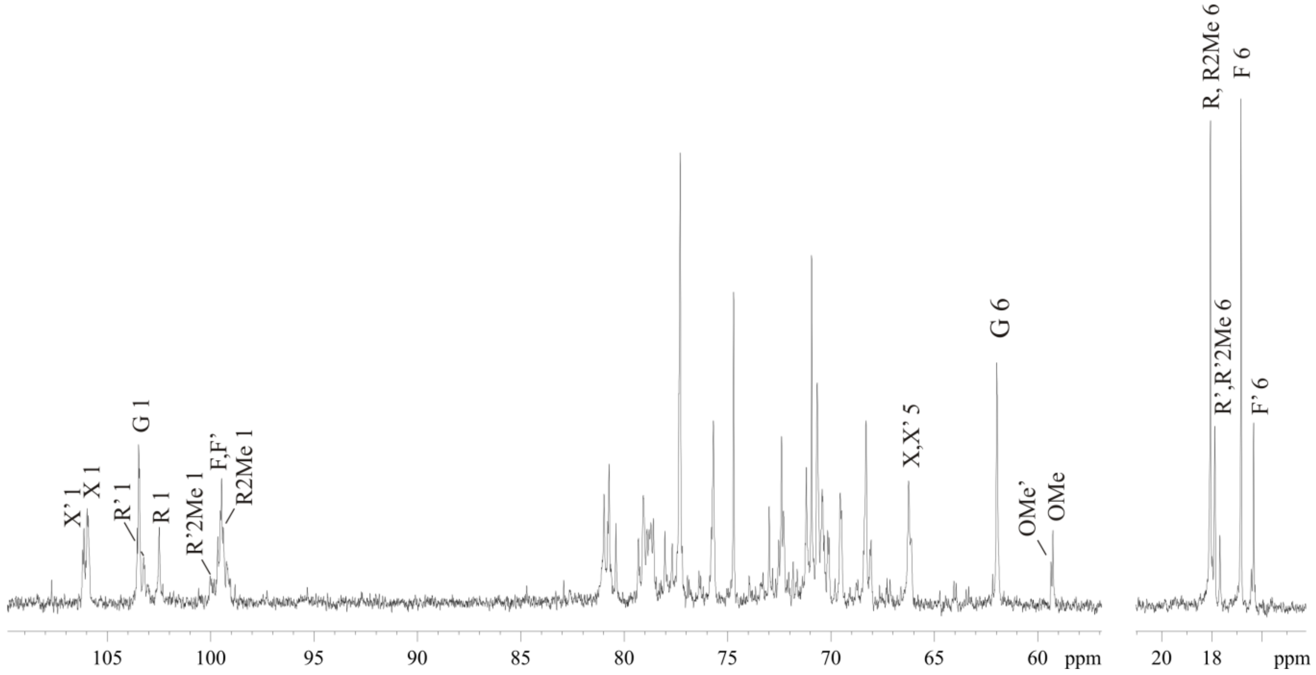

Figure S2: ^{13}C NMR spectrum of the OPS from *A. halopraeferens* Au4. Arabic numerals refer to carbons in sugar residues as denoted in Scheme S1.

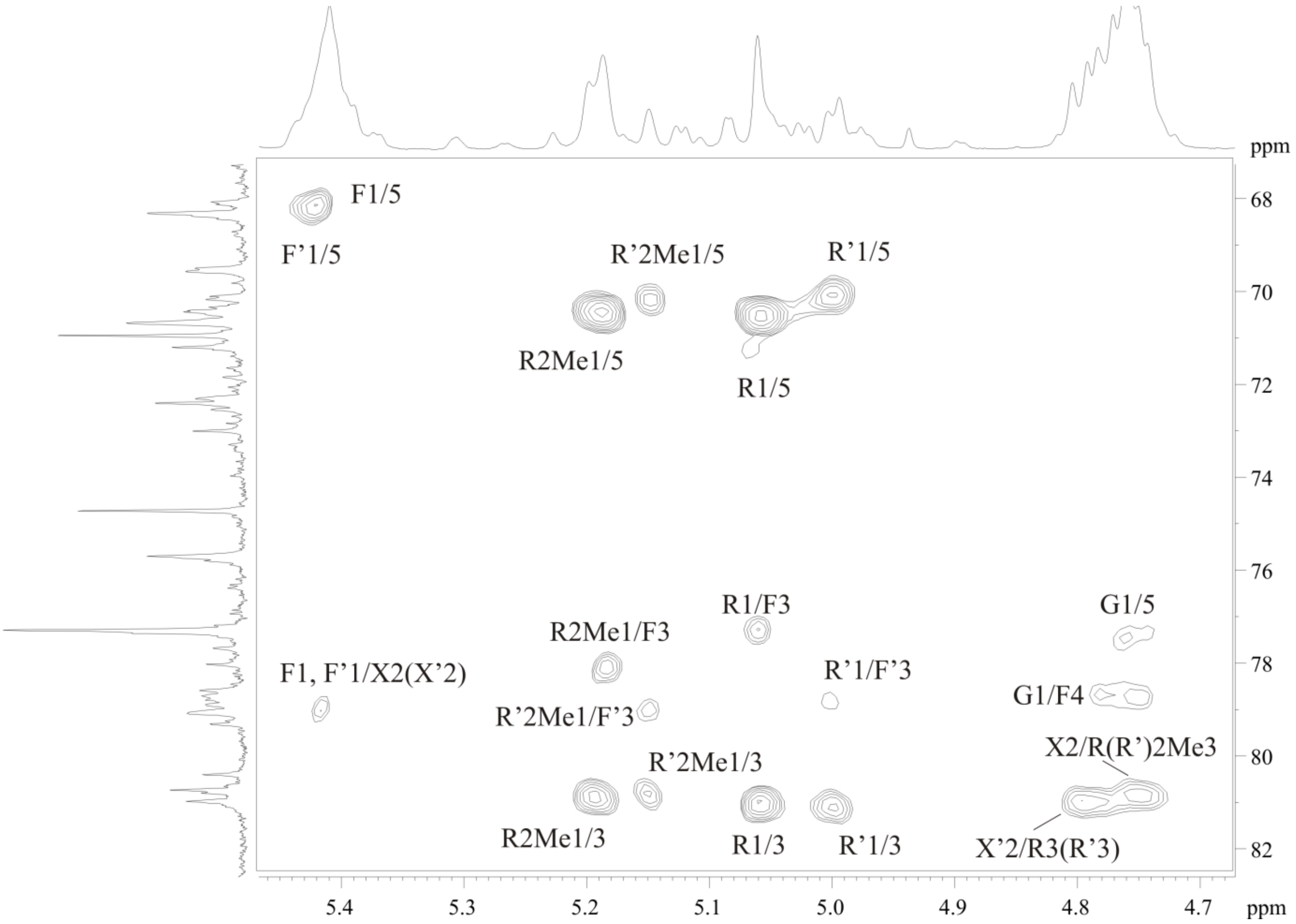

Figure S3: Fragment of the ^{1}H, ^{13}C HMBC spectrum of the OPS from *A. halopraeferens* Au4. Arabic numerals refer to C/H correlations in sugar residues as denoted in Scheme S1.

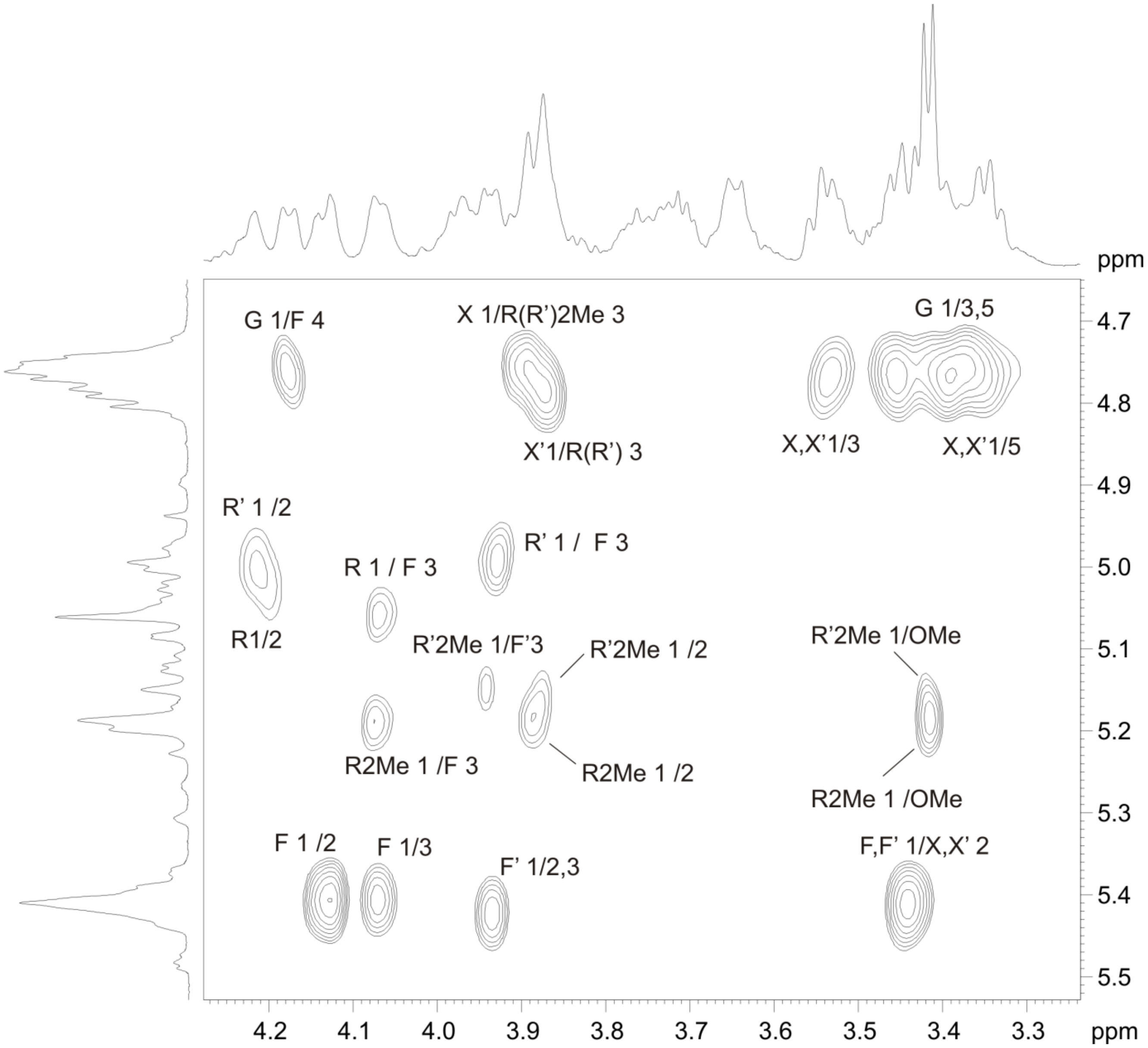

Figure S4: Fragment of the ^{1}H, ^{1}H ROESY spectrum of the OPS from *A. halopraeferens* Au4. Arabic numerals refer to C/H correlations in sugar residues as denoted in Scheme S1.

(R) (F)
α-L-Rha*p*-(1→3)-α-D-Fuc*p*-(1→2)-Gro **1**

(R2Me) (F)
α-L-Rha*p*-(1→3)-α-D-Fuc*p*-(1→2)-Gro **2**
2
|
OMe

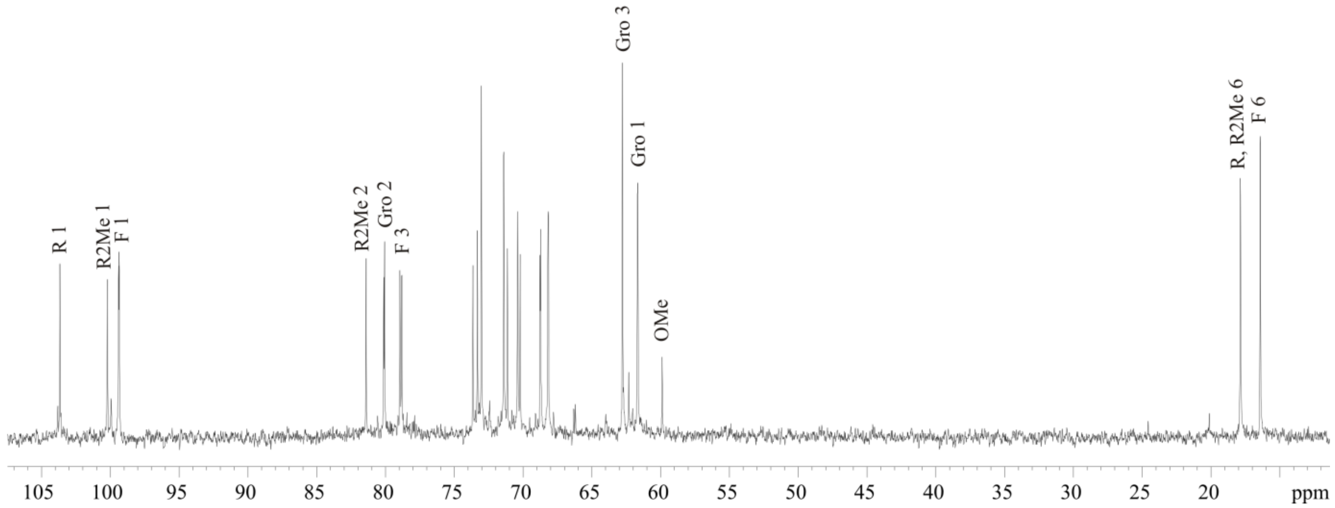

Figure S5: ^{13}C NMR spectrum of the mixture of the oligosaccharides **1** and **2** obtained after Smith degradation of the OPS from *A. halopraeferens* Au4. Arabic numerals refer to carbons in sugar residues as denoted.

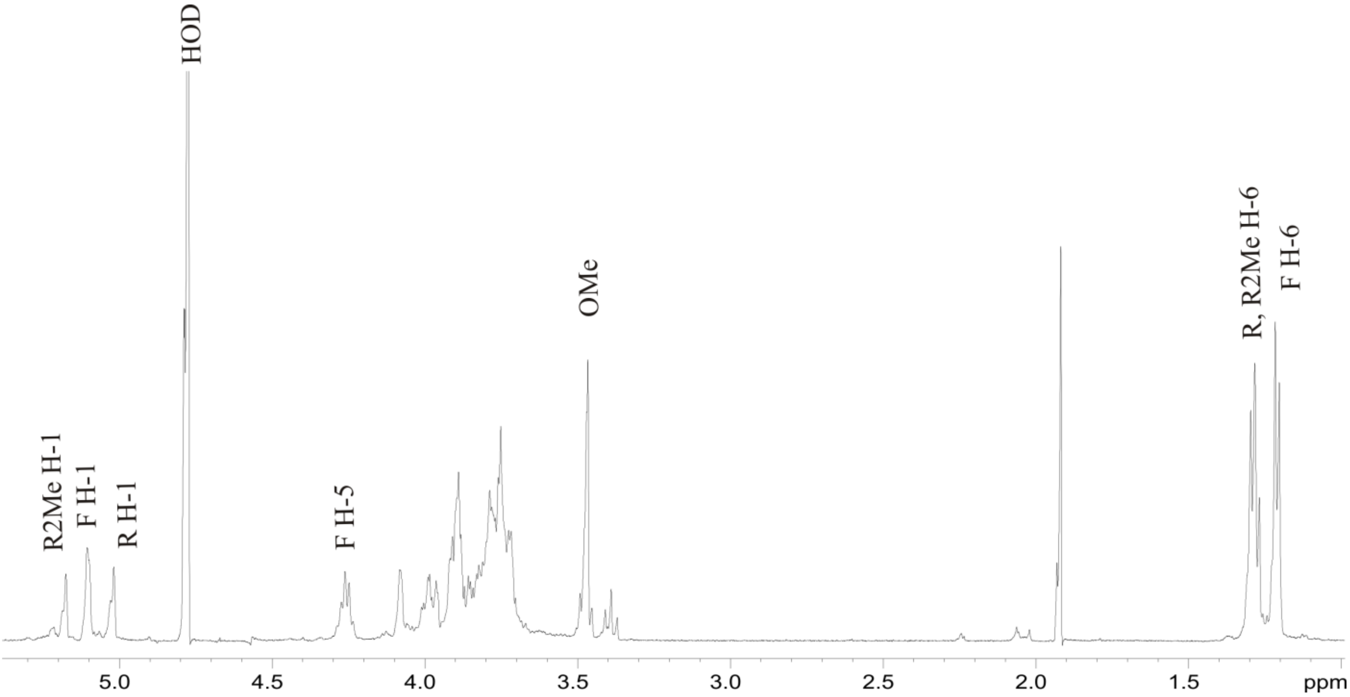

Figure S6: ^{1}H NMR spectrum of the mixture of the oligosaccharides **1** and **2** obtained after Smith degradation of the OPS from *A. halopraeferens* Au4. Arabic numerals refer to protons in sugar residues as denoted in Figure S5.

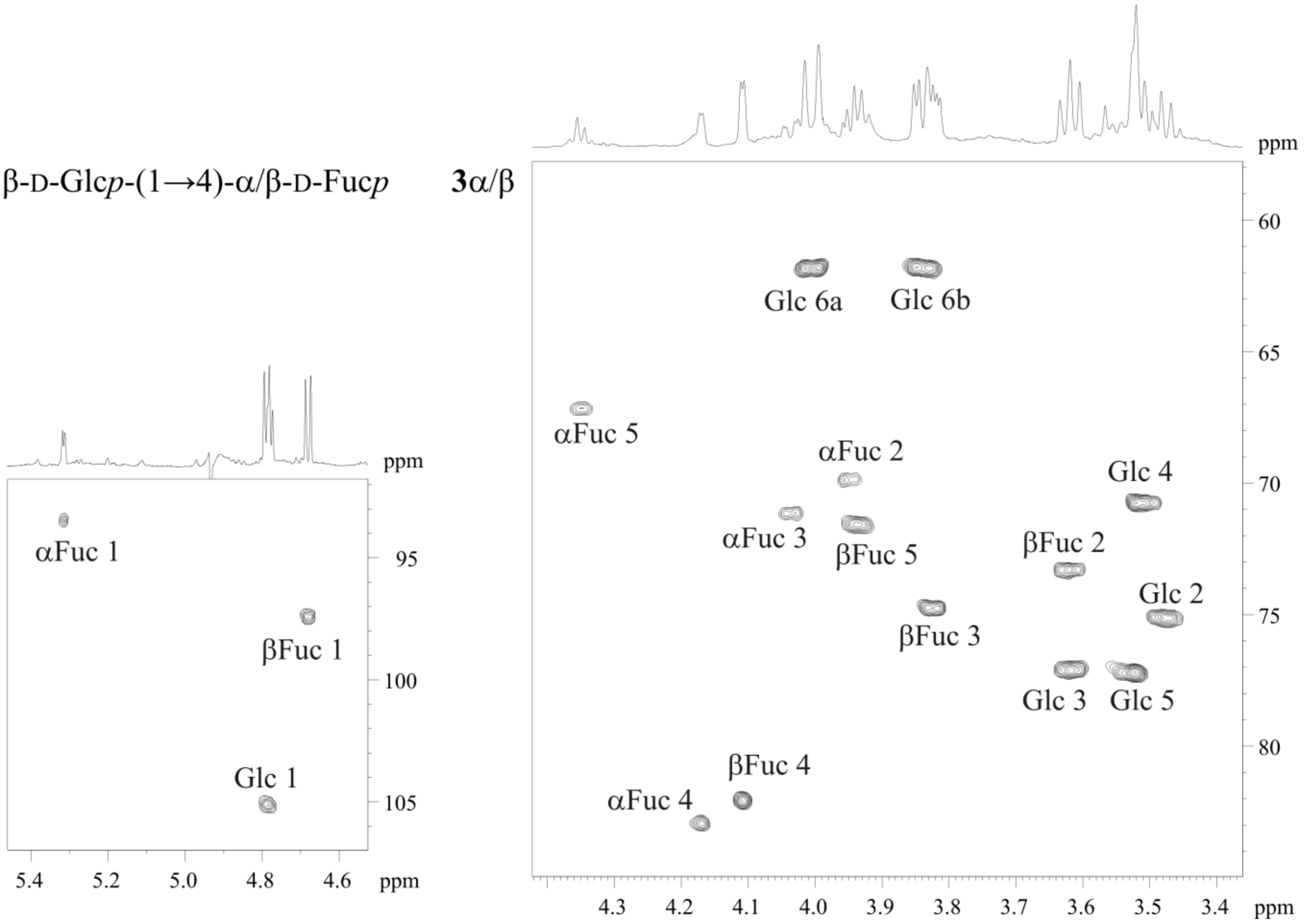

Figure S7: Fragments of the ^{1}H, ^{13}C HSQC spectrum of the disaccharide **3** obtained after solvolysis of the OPS from *A. halopraeferens* Au4. Arabic numerals refer to H, C pairs in sugar residues.
